# Effect of a nurse-led caring contact program on 90-day recurrence of nonsuicidal self-injury in youth

**DOI:** 10.1097/MD.0000000000048167

**Published:** 2026-04-03

**Authors:** Lei Tian, Qian Yong, Jun Fu

**Affiliations:** a Department of Psychiatry, Wuhan Mental Health Center, Wuhan City, Hubei Province, China; b Wuhan Children’s Hospital (Wuhan Maternal and Child Healthcare Hospital), Tongji Medical College, Huazhong University of Science & Technology, Wuhan City, Hubei Province, China; c Department of Nursing, General Hospital of Central Theater Command, Wuhan City, Hubei Province, China.

**Keywords:** adolescents, caring contact, nonsuicidal self-injury, nursing intervention, post-discharge follow-up, recurrence

## Abstract

Nonsuicidal self-injury (NSSI) is highly prevalent among adolescents and is associated with significant emotional dysregulation and elevated suicide risk. The post-discharge period, particularly the first 90 days, represents a high-risk window for relapse. Caring contact – a brief, supportive, nonclinical follow-up after discharge – has shown potential in reducing self-harm among adults, but evidence in adolescents with NSSI remains limited. This study aimed to evaluate the effectiveness of a nurse-led caring contact program in reducing 90-day NSSI recurrence and improving psychological outcomes among adolescents. A retrospective cohort study was conducted including 102 adolescents hospitalized for NSSI between January 2023 and December 2024. Participants were assigned to the intervention group (n = 52; structured caring contact at weeks 1, 2, 4, 8, and 12) or control group (n = 50; routine post-discharge care). Primary outcome was 90-day NSSI recurrence. Secondary outcomes included changes in depression (patient health questionnaire-9 item), anxiety (generalized anxiety disorder 7-item scale), and perceived social support (perceived social support scale). Logistic regression was used to identify independent predictors of recurrence. Nurse-led caring contact substantially reduces short-term NSSI recurrence and improves emotional symptoms and perceived social support in adolescents following psychiatric hospitalization. As a low-cost, scalable intervention, caring contact should be considered for integration into routine post-discharge management to enhance continuity of care and prevent early relapse in this high-risk population. The 90-day recurrence rate was significantly lower in the intervention group compared with controls (21.15% vs 42.00%, χ^2^ = 5.17, *P* = .023). The intervention group showed greater reductions in patient health questionnaire-9 item and generalized anxiety disorder 7-item scale scores and a greater increase in perceived social support scale scores (all *P* < .001). In multivariable analysis, nurse-led caring contact remained an independent protective factor against recurrence (odds ratio = 0.38, 95% confidence interval: 0.16–0.91, *P* = .031).

## 1. Introduction

Nonsuicidal self-injury (NSSI) has emerged as a critical adolescent mental-health issue, defined as the deliberate infliction of physical harm without suicidal intent. Epidemiological data indicate that its lifetime prevalence in youth populations ranges from 17% to 30%, with higher rates consistently observed among female adolescents.^[1,2]^ NSSI is strongly linked to emotional dysregulation, adverse childhood experiences, and concurrent psychiatric symptoms – particularly depression and anxiety.^[3]^ Moreover, a substantial body of evidence shows that recurrent NSSI markedly increases the risk of future suicide attempts, underscoring the urgent need for early relapse-prevention strategies.^[4]^

The early post-discharge period represents a uniquely vulnerable window for recurrence. Studies have shown that up to half of adolescents who self-injure may relapse within the first 3 months after hospitalization, often due to inadequate coping skills, unstable family dynamics, or fragmentation of outpatient care.^[5,6]^ Although structured psychotherapies such as dialectical behavior therapy and cognitive behavioral therapy can attenuate self-harm risk, their accessibility and adherence remain limited for many adolescents, highlighting the necessity for interventions that are scalable, low intensity, and feasible in routine clinical settings.^[7]^

Caring contact interventions – brief, nonclinical expressions of concern delivered through letters, phone calls, or text messages – have gained renewed attention in recent years. Originating from Motto’s pioneering suicide-prevention work, this approach aims to maintain supportive connection and reduce perceived isolation among individuals at heightened risk.^[8]^ Contemporary evidence in adult populations suggests that caring contacts can reduce repeated self-harm and improve engagement with mental-health services.^[9]^ Adolescents, whose developmental needs are closely tied to interpersonal connection, may be particularly responsive to such supportive follow-up strategies.

Nurses occupy a central role in adolescent psychiatric care, serving as consistent care providers and maintaining therapeutic relationships that extend beyond hospitalization. Previous studies have demonstrated that nurse-led interventions, including crisis follow-up and structured telephone support, can reduce self-harm behaviors among youth.^[10]^ Despite their theoretical promise, empirical data examining the effects of nurse-led caring contact specifically for adolescent NSSI remain limited. Therefore, the present study sought to determine whether a structured nurse-led caring contact program could reduce 90-day recurrence of NSSI following psychiatric discharge, and whether it may additionally influence depressive symptoms, anxiety, and perceived social support.

## 2. Materials and methods

### 2.1. Study design and setting

This study was approved by the Ethics Committee of General Hospital of Central Theater Command. This retrospective cohort study was conducted in our hospital. Medical records of adolescents diagnosed with NSSI between January 2023 and December 2024 were reviewed. This retrospective cohort study aimed to evaluate the effectiveness of a nurse-led caring contact program on the 90-day recurrence of NSSI following hospital discharge.

### 2.2. Participants

#### 2.2.1. Inclusion criteria

Adolescents were eligible if they: were aged 12 to 18 years; met the Diagnostic and Statistical Manual of Mental Disorders, Fifth Edition (DSM-5) criteria for NSSI; 0 completed a standardized psychological assessment before discharge; had at least 1 reliable method of post-discharge contact (phone, short message service [SMS], or WeChat); and provided informed consent (patient and guardian).

#### 2.2.2. Exclusion criteria

Patients were excluded if they: had comorbid psychotic disorders, bipolar disorder, or substance abuse; had severe cognitive impairment preventing follow-up; had incomplete baseline or follow-up data; and refused participation in post-discharge follow-up.

#### 2.2.3. Sample size

Group allocation was determined by the timing of program implementation. Patients discharged before the introduction of the structured caring contact program received routine post-discharge care (control group), whereas those discharged after implementation received the nurse-led caring contact intervention (intervention group). No randomization procedure was performed.

### 2.3. Interventions

#### 2.3.1. Control group (routine psychological nursing)

Patients in the control group received standard inpatient psychological nursing care, including: emotional support and crisis stabilization; health education regarding NSSI risk factors and coping strategies; routine discharge guidance; and referral for outpatient psychological services if needed. No structured post-discharge follow-up was provided beyond routine clinical appointments.

#### 2.3.2. Intervention group (nurse-led caring contact program)

In addition to routine care, the intervention group received a structured caring contact program delivered by a trained psychiatric nurse. Caring contacts were initiated via telephone, SMS, or WeChat at 1, 2, 4, 8, and 12 weeks after discharge. Each caring contact included: brief expressions of care and support (e.g., “We’re thinking of you. How have you been coping recently?”); reinforcement of healthy coping strategies; encouragement to seek help earlier if emotional distress increased; and monitoring for warning signs of relapse or deterioration. Importantly, caring contacts avoided clinical counseling, treatment advice, or emergency instructions; instead, they emphasized warmth, concern, continuity, and supportive connection. All follow-up interactions were documented in the nursing communication log.

### 2.4. Data collection procedures

Two trained research nurses independently extracted baseline characteristics, clinical histories, follow-up notes, and recurrence information from electronic medical records. Discrepancies were resolved by a senior psychiatric clinician. All personal-identifiable information was removed prior to analysis.

### 2.5. Statistical analysis

Statistical analyses were performed using SPSS version 26.0 (IBM Corp., Armonk). Continuous variables were presented as mean ± standard deviation and compared with independent-samples *t* tests. Categorical variables were analyzed using χ^2^ tests or Fisher’s exact test when appropriate. Variables with *P* < .10 in univariate analysis were entered into a multivariate logistic regression model to identify independent predictors of NSSI recurrence. Statistical significance was defined as *P* < .05 (2-tailed).

### 2.6. Ethical considerations

This study was approved by the Institutional Ethics Committee of the hospital. As this research was retrospective in nature, waiver of informed consent was granted for record review; however, all caring contact participants and guardians provided informed consent for follow-up communication. All procedures complied with the Declaration of Helsinki.

## 3. Results

### 3.1. Baseline characteristics

A total of 102 adolescents diagnosed with NSSI were included, comprising 50 in the control group and 52 in the intervention group. The mean age was 15.6 ± 1.7 years, and 78.4% were female. There were no statistically significant differences between groups in terms of age, gender, family structure, or baseline psychological scores (patient health questionnaire-9 item [PHQ-9], generalized anxiety disorder 7-item scale [GAD-7], and perceived social support scale [PSSS]; all *P* > .05), indicating good baseline comparability (Table 1).

**Table 1:** Baseline characteristics of adolescents with nonsuicidal self-injury (NSSI)

Variable	Control group (n = 50)	Intervention group (n = 52)	χ^2^/*t*	*P* value
Age (yr, mean ± SD)	15.7 ± 1.8	15.6 ± 1.7	0.28	.781
Female, n (%)	39 (78.0%)	41 (78.8%)	0.01	.938
Only child, n (%)	23 (46.0%)	25 (48.1%)	0.04	.841
Single-parent family, n (%)	11 (22.0%)	9 (17.3%)	0.37	.542
Duration of NSSI (mo, median [IQR])	8 (5–13)	7 (5–12)	0.65	.518
PHQ-9 baseline score	11.42 ± 3.35	10.96 ± 3.28	0.67	.506
GAD-7 baseline score	10.21 ± 3.68	9.78 ± 3.46	0.60	.550
PSSS baseline score	43.18 ± 5.74	43.92 ± 6.03	0.64	.524

GAD-7 = generalized anxiety disorder 7-item scale, IQR = interquartile range, NSSI = non-suicidal self-injury, PHQ-9 = patient health questionnaire-9 item, PSSS = perceived social support scale, SD = standard deviation.

### 3.2. Comparison of 90-day recurrence

**Figure 1.**
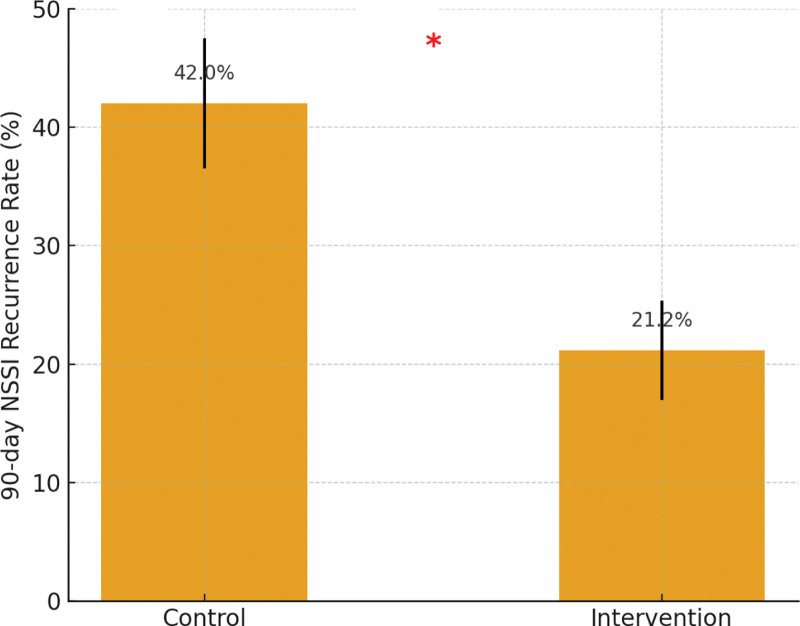
Comparison of 90-day recurrence rate between groups. NSSI = nonsuicidal self-injury.

During the 90-day follow-up, the recurrence rate of NSSI was significantly lower in the intervention group (21.15%) than in the control group (42.00%; *χ*^*2*^ = 5.17, *P* = .023). The results suggest that the nurse-led caring contact program effectively reduced short-term relapse after discharge (Table 2 and Fig. 1).

**Table 2:** Comparison of 90-day NSSI recurrence rate between groups.

Outcome	Control group (n = 50)	Intervention group (n = 52)	χ^2^	*P* value
NSSI recurrence, n (%)	21 (42.00%)	11 (21.15%)	5.17	.023
No recurrence, n (%)	29 (58.00%)	41 (78.85%)	–	–

NSSI = non-suicidal self-injury.

The bar chart shows a markedly lower recurrence rate in the intervention group (21.15%) compared with the control group (42.00%). The red asterisk (*) indicates a statistically significant difference (*P* < .05).

### 3.3. Psychological assessment outcomes

**Figure 2.**
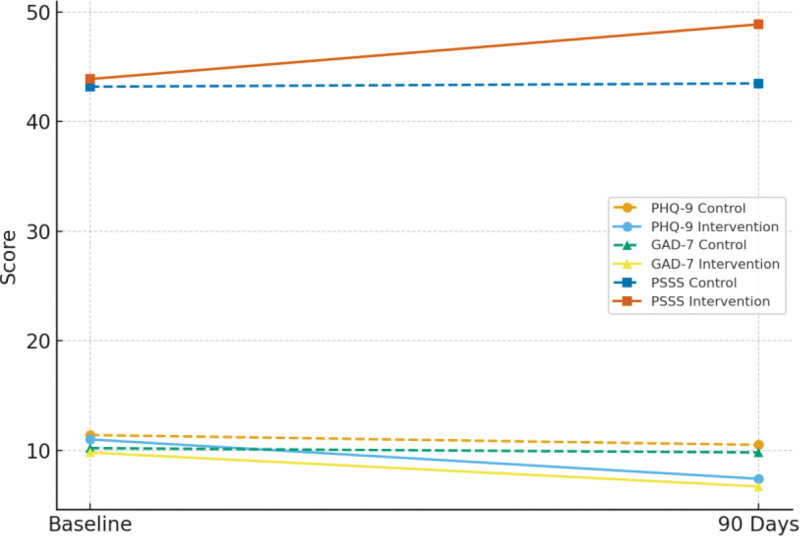
Changes in PHQ-9, GAD-7, and PSSS scores before and after intervention. GAD-7 = generalized anxiety disorder 7-item scale, PHQ-9 = patient health questionnaire-9 item, PSSS = perceived social support scale.

At the 90-day follow-up, both groups exhibited reductions in depression (PHQ-9) and anxiety (GAD-7) scores, and improvement in perceived social support (PSSS). However, the intervention group demonstrated significantly greater improvements in all 3 indicators compared with the control group (all *P* < .001; Table 3 and Fig. 2).

**Table 3:** Comparison of PHQ-9, GAD-7, and PSSS scores at 90 days post-intervention.

Scale	Control group (mean ± SD)	Intervention group (mean ± SD)	*t*	*P* value
PHQ-9	10.48 ± 3.21	7.36 ± 2.82	5.16	<.001
GAD-7	9.82 ± 3.57	6.74 ± 3.05	4.73	<.001
PSSS	43.52 ± 6.18	48.93 ± 5.75	−4.54	<.001

GAD-7 = generalized anxiety disorder 7-item scale, PHQ-9 = patient health questionnaire-9 item, PSSS = perceived social support scale, SD = standard deviation.

The line chart shows that PHQ-9 and GAD-7 scores decreased more markedly in the intervention group, while PSSS scores increased substantially. The trajectories reflect better emotional recovery and social support in the nurse-led caring contact group.

### 3.4. Multivariate logistic regression analysis

Multivariate logistic regression was conducted including age, sex, baseline PHQ-9, GAD-7, and group allocation. After adjustment, the nurse-led caring contact intervention remained an independent protective factor against NSSI recurrence (odds ratio = 0.38, 95% confidence interval: 0.16–0.91, *P* = .031; Table 4).

**Table 4:** Multivariate logistic regression analysis of factors associated with 90-day NSSI recurrence

Variable	β	SE	OR (95% CI)	*P* value
Age	0.05	0.08	1.05 (0.90–1.23)	.526
Female (vs male)	0.27	0.54	1.31 (0.45–3.84)	.619
PHQ-9 baseline	0.10	0.06	1.11 (0.98–1.25)	.103
GAD-7 baseline	0.08	0.07	1.08 (0.94–1.24)	.252
Caring contact (yes vs no)	−0.97	0.45	0.38 (0.16–0.91)	.031

CI = confidence interval, GAD-7 = generalized anxiety disorder 7-item scale, OR = odds ratio, PHQ-9 = patient health questionnaire-9 item, SE = standard error.

## 4. Discussion

This study found that participation in a nurse-led caring contact program was associated with a significantly lower 90-day recurrence rate of NSSI among adolescents following psychiatric discharge. However, these findings must be interpreted with caution due to important methodological limitations.

First and foremost, this study employed a retrospective, non-randomized cohort design. Group allocation was determined by the timing of program implementation rather than by random assignment, which introduces potential selection bias and temporal confounding. Although baseline characteristics were statistically comparable between groups, unmeasured confounders – such as family support dynamics, illness severity, prior psychotherapy exposure, and outpatient adherence – may have influenced recurrence risk. Therefore, causal inference cannot be established, and the observed association should not be interpreted as definitive evidence of efficacy.^[11]^

Additionally, the time-based allocation strategy may introduce secular trend bias, as broader contextual factors during the implementation period could have contributed to outcome differences. The intervention’s beneficial effects are likely mediated through enhanced social connectedness and improved emotional regulation. Prior research underscores that interpersonal stress, invalidation, and social isolation are key drivers of self-injurious behavior, whereas supportive communication can interrupt maladaptive emotional cascades.^[12,13]^ In this study, the intervention group exhibited greater improvements in depressive symptoms, anxiety, and perceived social support, suggesting that caring contact may reinforce healthy coping strategies and strengthen relational protective factors. These findings are consistent with theoretical models – such as the interpersonal theory of suicide and the emotional cascade model – which emphasize the central role of social connectedness in mitigating self-injury risk.^[14]^

Another plausible mechanism is that caring contacts function as early behavioral prompts that facilitate timely help-seeking before emotional crises escalate. Adolescents frequently encounter barriers to seeking support, including stigma, low distress tolerance, and difficulty recognizing deterioration.^[15]^ Structured follow-up at predetermined intervals (weeks 1, 2, 4, 8, and 12) may normalize help-seeking, encourage proactive communication, and provide opportunities to detect emerging risks. Similar scheduled-contact frameworks have been shown to support ongoing engagement and reduce crisis episodes in youth mental-health populations.^[16-18]^

Despite promising results, several limitations warrant consideration. The retrospective design precludes definitive causal inference, and unmeasured confounders – such as family environment, psychiatric severity, or prior psychotherapy – may influence outcomes. Self-reported recurrence may underrepresent actual event frequency. Furthermore, the optimal modality, frequency, and content of caring contact are yet to be determined. Future research should involve multicenter randomized controlled trials, explore modality-specific effects (SMS, telephone, digital platforms), assess long-term sustainability, and incorporate qualitative evaluations of adolescents’ experiences with caring contact.^[19,20]^ Overall, this study supports integrating nurse-led caring contact into routine post-discharge care for adolescents with NSSI as a feasible, low-cost strategy to reduce relapse and enhance psychosocial resilience.

## 5. Conclusion

This study demonstrates that nurse-led caring contact is a simple, scalable, and effective strategy to reduce early recurrence of NSSI in adolescents and to improve emotional symptoms and perceived social support following discharge. Incorporating caring contact into routine aftercare may enhance continuity of care and strengthen relapse prevention in this high-risk population. Future multicenter randomized controlled trials are needed to confirm these findings and to determine the optimal modality and frequency of caring contact interventions.

## Author contributions

**Conceptualization:** Lei Tian, Qian Yong, Jun Fu.

**Data curation:** Lei Tian, Qian Yong, Jun Fu.

**Formal analysis:** Lei Tian, Qian Yong, Jun Fu.

**Funding acquisition:** Lei Tian, Qian Yong, Jun Fu.

**Investigation:** Lei Tian, Qian Yong.

**Writing – original draft:** Lei Tian, Qian Yong, Jun Fu.

**Writing – review & editing:** Lei Tian, Qian Yong, Jun Fu.
